# Cell Proliferation in the Adult Chicken Hippocampus Correlates With Individual Differences in Time Spent in Outdoor Areas and Tonic Immobility

**DOI:** 10.3389/fvets.2020.00587

**Published:** 2020-08-26

**Authors:** Elena A. Armstrong, Bernhard Voelkl, Sabine Voegeli, Sabine G. Gebhardt-Henrich, Jonathan H. Guy, Victoria Sandilands, Tim Boswell, Michael J. Toscano, Tom V. Smulders

**Affiliations:** ^1^Centre for Behaviour and Evolution, Newcastle University, Newcastle upon Tyne, United Kingdom; ^2^Biosciences Institute, Newcastle University, Newcastle upon Tyne, United Kingdom; ^3^Centre for Proper Housing: Poultry and Rabbits (ZTHZ), University of Bern, Bern, Switzerland; ^4^School of Natural and Environmental Sciences, Newcastle University, Newcastle upon Tyne, United Kingdom; ^5^Department of Agriculture, Horticulture, and Engineering Science, SRUC, Edinburgh, United Kingdom

**Keywords:** hippocampal formation, avian brain, adult neurogenesis, free-range laying hens, individual differences, animal welfare, *Gallus gallus domesticus*

## Abstract

Access to outdoor areas is provided as a means of enhancing welfare in commercial systems for laying hens (*Gallus gallus domesticus*), but substantial individual differences exist in their proportional use. Baseline cell proliferation levels of Adult Hippocampal Neurogenesis (AHN) have been associated with individual differences in reactive vs. proactive coping style, and in both mammals and birds, AHN is upregulated by positive experiences including environmental enrichment and exercise. We thus sought to explore whether individual differences in use of outdoor areas and in tonic immobility responses (indicative of fearfulness) were associated with hippocampal cell proliferation and neuronal differentiation. Radio frequency identification technology was used to track the ranging behavior of 440 individual focal hens within a commercially-relevant system over a 72-days period, after which tonic immobility durations were measured. Following hippocampal tissue collection from 58 focal hens, proliferation and neuronal differentiation were measured through quantitative PCR for proliferating cell nuclear antigen (PCNA) and doublecortin mRNA, respectively. Individual differences in tonic immobility duration positively correlated with PCNA expression over the whole hippocampal formation, while greater time spent in outdoor areas (the grassy range and stone yard) was associated with higher proliferation in the rostral subregion. Basal proliferation in the chicken hippocampal formation may thus relate to reactivity, while levels in the rostral region may be stimulated by ranging experience. Doublecortin expression in the caudal hippocampus negatively co-varied with time on the grassy range, but was not associated with tonic immobility duration. This suggests that ranging outside may be associated with stress. Within laying hen flocks, individual differences in hippocampal plasticity thus relate to coping style and use of external areas.

## Introduction

Within commercial flocks of laying hens, variation between individuals may be associated with differing experience and overall welfare. For example, many systems provide access to outdoor areas as a form of enrichment, which expands freedom of movement, behavioral repertoire, exploration, and foraging opportunities for hens, beyond those already afforded by the barns (1, 2). Outdoor ranges also offer an environment of greater unpredictability than the barn interior, where conditions are tightly controlled (3). However, there is substantial variation in the extent to which individual hens use these external areas. Use of radio frequency identification (RFID) tracking within flocks consistently highlights distinct subgroups, wherein a proportion of hens access the range daily, while others seldom or never venture outside (4–7). Factors underlying this variation in ranging propensity are not yet understood, but may relate to aspects of personality, defined as consistent inter-individual differences in behavior (8).

A well-characterized dimension of animal personality is the tendency to adopt an active (or proactive) vs. passive (or reactive) behavioral strategy when challenged (9), also referred to as a coping style (10). Reactive/passive individuals are predisposed toward displaying a freezing-type fear response as opposed an active fight or flight response (11), and are thus more easily induced into immobility and remain in this state longer (9, 12). Consequently, individual differences in reactivity for hens may be reflected in their durations of tonic immobility (TI): a catatonic-like freezing response induced by brief physical restraint in an upturned position (13). Consistent with a personality trait, variation in duration of the TI response is heritable (14, 15). In line with freezing less, proactive individuals are more prone to exploration (16, 17).

Behavioral strategy also relates to individual differences in speed vs. accuracy during learning, with proactive individuals acquiring simple novel tasks more quickly (18, 19). For example, black-capped chickadees (*Poecile atricapillus*) that readily enter a novel environment are faster to learn an acoustic discrimination task (20). In hens, proactive behavior has been shown to predict predisposition to use the outdoor range. When tested before any range access, pullets from an enriched rearing environment that were quickest to reach T-maze success (presumably pro-active) also proceeded to visit the range most frequently over the 4 successive weeks (3). However, while proactive individuals tend to maintain rigid, routine-like behavior, reactive individuals are more sensitive to changes in the environment/task requirements and display enhanced behavioral flexibility (10, 21, 22). Rats selectively bred for their ability to learn new configurations in a maze task were more susceptible to TI and slower to explore a novel environment than those bred for low maze performance (23). In birds, more explorative adult red junglefowl (19) and black-capped chickadees (24) are slower at reversal learning, while behavioral flexibility is positively correlated with fearfulness in junglefowl chicks (25). Moreover, low ranging broiler chickens improved in accuracy of spatial discrimination between trials of a memory task occurring on the same day, whereas higher rangers behaved inflexibly and did not alter their performance on the second trial (26). Compared to higher rangers, chickens that ranged less were also better at inhibiting their behavior by detouring to the sides of a transparent cylinder to access a food reward, rather than pecking the cylinder walls (27).

Such individual differences in behavior may be reflected by variation in neural plasticity. Plasticity has been defined as the reciprocal interaction between brain structure and function, and forms the neurobiological basis of individuality (28). A site of notable post-developmental plasticity in the mammalian brain is the hippocampus, wherein new neurons continue to be produced and functionally integrated into the dentate gyrus subfield (29–32) through a process called Adult Hippocampal Neurogenesis (AHN). AHN has several stages: (i) proliferation of progenitor cells; (ii) migration and neural differentiation; (iii) maturation of immature neurons; and (iv) functional integration of new mature neurons into the pre-existing neural circuitry (33, 34). The various stages of AHN can be quantified using different markers. Proliferating cell nuclear antigen (PCNA) is expressed by actively dividing cells (35), while doublecortin (DCX) is a microtubule-associated protein expressed by both proliferative (type-2b &−3) and post-mitotic differentiating neurons (33).

Interestingly, behavioral strategy is reflected in levels of proliferation in the hippocampus. Quantitative PCR indicated that Atlantic salmon (*Salmo salar*) characterized as reactive had a higher basal expression of PCNA mRNA in the hippocampal homolog than their proactive conspecifics (36). Furthermore, cell proliferation in rats that predominantly responded to a novel environment by freezing was twice that observed in proactive rats, while proliferating cell numbers positively correlated with durations of freezing on an individual level (37). The rigid and inflexible behavior displayed by proactive individuals has recently been linked to limitations in their neural plasticity (38). A causal role of newborn cells in flexible spatial behavior has been demonstrated through experimental suppression of neurogenesis. Mice with experimentally ablated AHN are impaired in learning a changed (reversed) goal location in a water maze (39), and in avoiding a rotating shock zone when this is added to a stationary zone learnt first (40). As such, an association between proliferation in the hippocampus and behavioral strategy may relate to the requirement of plasticity for flexible spatial behavior.

AHN is also sensitive to the environment and modulated by long-term experience. In the mammalian brain, AHN is stimulated by experiences associated with positive affect, including environmental enrichment (41), voluntary running exercise (42) and antidepressant treatment (43, 44), but suppressed by various forms of chronic negative stress [e.g., (45, 46)]. In line with a functional gradient across the longitudinal axis of the mammalian hippocampus (47), enrichment (48, 49), and exercise (50) preferentially upregulate AHN in the dorsal mouse dentate gyrus, while chronic stress suppresses it in the ventral region (51). The increase in AHN due to environmental enrichment is also typically accompanied by a decrease in anxiety-like behavior in mice (52).

Though the avian hippocampal formation (HF) differs from the mammalian structure in cytoarchitecture and notably lacks a dentate gyrus, they are homologous, and functional similarities are evident in domains including navigation, spatial memory, and modulation of the glucocorticoid stress response (53–55). AHN levels in birds are similarly stimulated by environmental enrichment and complexity (56, 57), but downregulated by sources of chronic stress, including captivity, food restriction, constant light, unpredictable chronic mild stress, and keel bone fractures (58–62). A homologous functional gradient may also exist in the avian HF (63, 64), wherein the rostral subregion is equivalent to the dorsal rodent region and the caudal avian region is the ventral rodent homolog.

In the present study, RFID tags were used to track individual ranging behavior in terms of the proportional time that hens spent in four distinct areas: (1) the barn, (2) an adjoining covered wintergarden, (3) an adjacent uncovered stone yard, and (4) a large, grassy range. This set-up may facilitate separation of the implications that various aspects of the environment have for hen behavior and AHN. For example, the wintergarden provides fresh air but cover from rain, both the stone yard and range are exposed to the elements and to predators, and the range alone provides grass. Ranging a greater distance from the barn has been positively associated with welfare parameters in broiler chickens (65). At the end of the study, TI durations were measured and hippocampal expression of PCNA and DCX was quantified. Our research group has previously established that transcription of the DCX gene in the mouse hippocampus reflects DCX-immunoreactive cell densities under control and enriched housing conditions (49).

If individual differences in AHN relate to personality type, we would predict pro-active hens with shorter TI times to be more likely to explore the range, and AHN should co-vary negatively with ranging and positively with TI. However, if ranging experience upregulates AHN while reducing anxiety, individual differences in AHN should correlate positively with time on the range but negatively with durations of TI. Based upon putative subregional specialization in the HF, in the latter scenario, cognitive enrichment arising from broader ranging would be predicted to correlate most strongly with AHN in the rostral HF, while a negative relationship between anxiety (TI) and AHN may be evident especially in the caudal region. In the former scenario, AHN should correlate positively with TI time throughout the entire HF. This work represents a first exploration of the potential associations between hippocampal plasticity, ranging behavior and coping style in domestic chickens.

## Materials and Methods

### Animals and Facilities

Experimental use of the animals was approved by the Bern Kantonal Authority (BE-46/16) and the Animal Welfare and Ethical Review Body at Newcastle University (Project ID #549), and procedures complied with Swiss regulations regarding their treatment. Standard commercial protocols were followed, including *ad-libitum* access to food and water. Following on-site rearing [detailed in (66)], 17 weeks old Brown Nick (H&N International) laying hens were transferred to a commercial laying hen house at the Aviforum (Zollikofen, Switzerland). Only one of the barn's two halves was used for the present study, wherein pens were equipped with a system that allowed the tracking of individual animals. The four study pens (each 12.9 m^2^) contained a Rihs Bolegg II commercial aviary system (Krieger AG, Ruswil, Switzerland) with a stocking density of 9.33 hens/m^2^. The aviary structure and group nests lined one wall, and the floor of the barn was covered with 10 cm of wood shavings. The aviary was 2.40 m high and consisted of three tiers, with integrated equipment comprising: a manure belt, feeding chain, and nipple drinkers within the lowest tier; a manure belt within the middle tier; and a feeding chain and nipple drinkers within the highest tier. Plastic mushroom-shaped perches were provided on the lowest and highest tiers and plastic platforms to move between tiers were provided along both aviary sides (30 cm in width and at 70 cm height from the floor). Nest entries were square plastic grids (size 2.5 × 5 cm). External to the barn were three separate areas: a wintergarden, stone yard and grassy range, each linked at a single location (pophole or gate) to facilitate sequential movement of birds when open, but closed to limit access as required by the management protocol. Fencing between pens maintained divided populations within all (internal and external) areas. Adjacent to the barn interior, birds had access to the winter-garden (~17.55 m^2^ per pen), which was entirely covered by a solid roof and surrounded by wire mesh on the sides and in between pens. The floor of the winter-garden was lined with a thin layer of wood shavings of the same type provided within the barn, and the area was equipped with nipple drinkers and perches. A manually-operated pophole separated the winter-garden from an uncovered yard area (~88 m^2^ per pen) which was lined with small stones and enclosed by a fence. Beyond a gate in the fence surrounding the stone yard was the “free-range:” an open, grassy pasture with an average size of 288 m^2^ per pen. The grass was routinely mowed, and access was restricted during periods of dry weather to ensure it was maintained. Upon introduction to the barn, 355 hens were placed into each of the four pens, and 110 randomly selected birds per pen were fitted with an RFID transponder (Hitag S 2,048 bits, 125 kHz) attached to an adjustable leg band (IDs, Roxan, Scotland). Artificial light was provided in the barn from 200 to 1,700 h, with transitional phases of five min beginning at 200 h and 15 min beginning at 1,645 h. Natural daylight was provided from 800 to 1,630 h through windows controlled by curtains. To allow hens to acclimatize to the barn interior, they were kept inside for the 1st week. Subsequent access to the wintergarden, stone yard, and range was first provided one, 2 and 4 weeks after population, respectively. For the subsequent 5-months period (June 7–October 16, 2016), birds were permitted weather-dependent voluntary daily access to the external areas. Antennae were positioned on either side of the transition points (popholes/gates) connecting two areas and RFID transponders recorded the date and time of each zone-transition made. Records permitted calculation of the time spent in each area [as in (4)], but not distances traveled within them. At the conclusion of the daily period for which birds were provided outside access, those in other areas were encouraged back into the barn interior. At 42 weeks of age, a roughly equal number of tagged birds from each pen were haphazardly selected for sampling of hippocampal tissues and behavioral assessment (total *n* = 58). Loss of samples from five birds during molecular biology processing resulted in a final sample size of 53 birds.

### Ranging Quantification

Daily observations started at the time the range was opened and stopped when the range was closed. Based upon weather conditions, daily opening times varied between 7:50 and 13:50 and closing times ranged from 14:20–16:55, though hens were typically allowed to range until 16:30 each day. On days that behavioral testing, management protocols (vaccinations) or poor weather required restricted access to outdoor areas, access was intentionally restricted equally across all pens. The percentage of time spent in each area by each hen was calculated based on the remaining observation period of 72 days, wherein the range was accessible for an average of 6 h 58 min per day. There were some instances of loss of antenna signal coverage, generally caused by birds moving too quickly for detection. This meant that not all transitions were recorded. However, data checks confirmed accurate recording of each animal's movement and location patterns (e.g., sequential progression through areas rather than “jumps” to more distal ones). On average, the location of a bird was recorded for 83% of the time the range was open (mean coverage rate, IQR 69–96%). Proportions of available time spent in each area by hens were calculated based only upon times wherein their individual zonal locations were transmitted, leaving an average of 5 h 47 min tracked per day. As missed recordings were distributed evenly over all areas, while the actual times that birds spent outdoors/on the range may have been higher than observed, the proportional times analyzed should not have been affected. Detailed data regarding movement of hens between the barn and external areas is reported elsewhere (66).

### Tonic Immobility

As part of related experimental evaluations [reported elsewhere: (67)], collection of final measurements spanned a four-day period in which all hens were prevented from leaving the barn. On each day, hens from a single pen were transported from their home pen to another barn on-site for the measurement of TI, which occurred shortly before tissue collection. To induce immobility, hens were placed on their backs on a holding frame, with a light pressure applied to the breast. After pressure was released, the latency until the hen righted itself was timed using a stopwatch. The same observer (SGH) conducted all TI tests. If immobility was not successfully induced (i.e., the bird started to move within 3 s of removal of pressure), the procedure was repeated up to three times. Where immobility was not induced after the final attempt, the hen received a latency of 0 s. If a hen remained immobile for 300 s, they were ascribed this value as the maximum latency and the test was terminated.

### Tissue Collection

Shortly after TI measurement, animals (*n* = 58) were killed via intravenous injection with pentobarbital (Esconarkon, 0.3 ml/hen due to similar weights). Immediately thereafter, brains were removed from the skull, placed into 0.1 M phosphate-buffered saline in a Petri dish and divided along the longitudinal fissure with a scalpel. From each hemisphere, the HF was dissected and divided midway across the rostrocaudal axis to produce two subsamples (rostral and caudal) containing equal amounts of tissue. This method constitutes a rough estimate of the boundary between the rostral and caudal HF, as the exact border between these putative functional subdivisions has yet to be clearly mapped out. The 4 HF samples collected from each hen were processed separately. Isolated HF regions were placed in sample tubes containing 1.5 ml of RNAlater^®^ Stabilization Solution (Thermo Fisher Scientific, UK) and refrigerated for 24 h before storage at −30°C.

### RNA Isolation and Reverse Transcription

RNA was extracted using TriSure reagent (Bioline, London, UK) and Lysing Matrix D tubes in a FastPrep Instrument (MP Biomedicals, Cambridge, UK). Purification of the RNA product combined with DNAse treatment was conducted with the Zymo Direct-zol™ RNA MiniPrep Kit (Cambridge Bioscience, Cambridge, UK), according to manufacturer's instructions. 2 μg RNA was reverse transcribed using the Tetro™ cDNA Synthesis Kit (Bioline, London, UK) for use in a quantitative real-time polymerase chain reaction (qPCR).

### Quantitative PCR

Gene specific primers were designed using the NCBI primer-BLAST tool and sequences are displayed in Table 1. As previously, the chicken lamin B receptor (LBR) gene was used as a control gene for normalization (68). Standards were produced by gel purification of PCR products using a MinElute gel extraction kit (Qiagen Ltd, Crawley, UK) and their concentration was measured with a NanoDrop spectrophotometer (Thermo Fisher Scientific, Loughborough, UK). Serial dilutions of standards were produced to create standard curves for qPCR quantification. qPCR reactions were run on a Bio-Rad model machine (Bio-Rad, California, USA). Reactions (20 μl) contained 5 μl of cDNA template together with 10 μl SYBR green master mix (No-ROX kit, Bioline, London, UK) and gene specific primers (400 nM). The manufacturer's instructions were followed for 3-step thermal cycling conditions. Samples were run in singlicate over three batches on a 96-well plate, each of which contained samples from animals across the spectrum of range use and was accompanied by a standard curve run in duplicate. No-template controls were also included. A melting curve analysis was performed to confirm specificity of reactions and efficiency values for the primers used ranged between 99.7 and 108.9%. Assays were analyzed using CFX-Manager software (Bio-Rad, California, USA).

**Table 1:** Gene-specific primers used for qPCR in tissue from the hippocampal formation.

**Gene**	**Accession**	**Orientation**	**Primer sequence (5**′**–3**′**)**	**Product length (bp)**
LBR	NM_205342	Forward	GGTGTGGGTTCCATTTGTCTACA	80
		Reverse	CTGCAACCGGCCAAGAAA	
PCNA	NM_204170.2	Forward	CAATGCGGATACGTTGGCTC	192
		Reverse	ACAGCATCACCAATGTGGCT	
DCX	NM_204335.3	Forward	AAGACGGCCCATTCGTTTGA	166
		Reverse	ATTTTTCGGGACCACAGGCA	

### Statistical Analysis

Analyses were conducted in IBM SPSS Statistics (v24). Linear mixed models (LMMs) were conducted to explore how the proportional times spent in each of the four areas (barn, wintergarden, stone yard, and range) by hens were related, while accounting for experimental pen as a random factor. Times in the intermediate areas (wintergarden and stone yard) were included separately as covariates while times in the extreme areas (barn and range) were dependent variables. Whether time in the wintergarden co-varied with time in the stone yard was also explored. Separate univariate ANOVAs were employed to determine whether TI durations and time spent in each area differed between experimental pens. A generalized linear model with a Poisson loglinear distribution was used to determine whether the number of attempts required to induce TI differed between pens. To explore whether ranging was related to TI durations, LMMs were conducted with TI duration as the dependent variable, pen as a random factor, number of attempts to induce TI as a fixed factor, and proportional time in each of the areas as covariates (over four individual models). Measured molar quantities of PCNA, DCX, and LBR mRNA were log(10)-transformed and, as the quantity of samples necessitated multiple qPCR runs, normalized using the Standard Score (Z_i_) within assays. Separate LMMs with unstructured covariance were conducted for PCNA and DCX, each with HF subregion (rostral/caudal) and sample (one per hemisphere) as repeated fixed factors, pen as a random factor and LBR expression in the same sample as a covariate. In individual models, percentage of time spent in each area was included as a covariate, as well as in their interaction with HF subregion. Two models explored whether TI was related to expression of each gene and included TI duration as a covariate, TI attempts as a fixed factor, and both variables in an interaction term with HF subregion. Where both TI duration and time in an area co-varied significantly with expression of the same gene, they were also included together in a single LMM to verify their explanation of independent proportions of the variance. The corrected gene expression values plotted in **Figures 2**, **3** comprise residual PCNA and DCX after accounting for LBR expression, rostrocaudal subregion, sample and pen in LMMs, as described above.

## Results

### Behavior

On average, the 53 focal hens spent the majority of available tracked time either in the barn or in the wintergarden (see Table 2). Proportional time in the wintergarden was therefore negatively correlated with time spent in the barn [*F*_(1, 50.9)_ = 199.7, *p* < 0.001, *B* = −1.212, SEM = 0.086]. One hen remained exclusively within the barn and never entered the wintergarden. Time spent in the wintergarden was positively correlated with time spent in the stone yard [*F*_(1, 50.5)_ = 11.06, *p* = 0.002, *B* = 0.161, SEM = 0.049], but did not predict time on the grassy range [*F*_(1, 50.5)_ = 1.04, *p* = 0.313, *B* = 0.052, SEM = 0.051]. Five hens never ventured outside (i.e., to the stone yard), while an additional three spent time in the stone yard but did not enter the grassy range. This meant that 45 hens (~85%) thus used all areas provided to some extent. Time spent in the stone yard was positively correlated with time on the range [*F*_(1, 50.5)_ = 18.12, *p* < 0.001, *B* = 0.494, SEM = 0.116].

**Table 2:** Descriptive statistics for proportions of available time (%) spent in each of the four areas of the housing system by focal hens (*n* = 53) over the tracked period.

	***N***	**Mean**	**Standard deviation**	**Median**	**IQR**	**Range**
Barn	53	51.31	26.61	41.00	51.64	11.81–100.00
Wintergarden	52	34.54	19.61	40.47	35.22	0.00–66.07
Stone yard	48	8.10	7.70	7.16	11.37	0.00–33.72
Range	45	6.05	7.30	2.28	7.90	0.00–28.28

*n indicates the number of birds that spent at least some time in each area*.

The mean number of daily transitions that hens made between areas correlated negatively with time in the barn [*F*_(1, 49.3)_ = 118.75, *p* < 0.001, *B* = −0.9753, SEM = 0.089] and positively with time in each of the three other areas [wintergarden: *F*_(1, 49.6)_ = 49.95, *p* < 0.001, *B* = 0.5895, SEM = 0.087; stone yard: *F*_(1, 49.3)_ = 46.84, *p* < 0.001, *B* = 0.2236, SEM = 0.033; range: *F*_(1, 49.5)_ = 20.72, *p* < 0.001, *B* = 0.1670, SEM = 0.037].

Behavior was compared between the four experimental pens. There was no difference in durations of TI between pens [*F*_(3,47)_ = 1.10, *p* = 0.358], and the number of attempts required to induce immobility did not differ (χ$\chi_{3}^{2}\;$ = 3.86, *p* = 0.277, see Table 3). Controlling for pen as a random factor, duration of the TI response did not differ with the number attempts to induce it [*F*_(2,44.9)_ = 0.92, *p* = 0.406].

**Table 3:** Descriptive statistics for durations of tonic immobility and number of attempts required to induce the state for hens from the four experimental pens.

	***N***	**Median (s)**	**IQR (s)**	**Range (s)**	***n* censored**	**Mean attempts**
Pen 1	17	269.3	226.4	12.3–300.0	7	1.27
Pen 2	10	290.0	155.4	60.1–300.0	5	2.30
Pen 3	11	261.4	172.5	78.0–300.0	4	1.82
Pen 4	15	109.7	282.8	6.5–300.0	3	1.93

*Censored times had the maximum duration of 300 s*.

**Figure 1:**
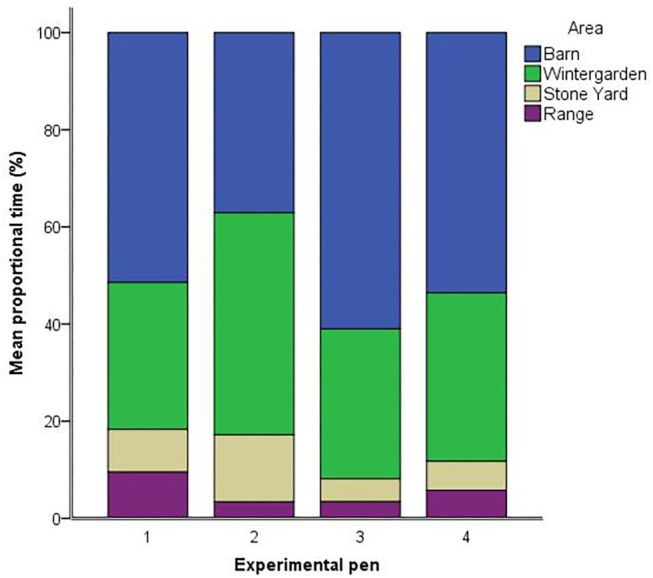
Mean proportions of available tracked time (%) spent in each of the four areas by hens in the four experimental pens.

In terms of ranging behavior, proportional time spent in the barn [*F*_(3,49)_ = 1.52, *p* = 0.222] and wintergarden [*F*_(3,49)_ = 1.53, *p* = 0.219] did not differ between pens. However, pens differed in the proportional time that hens spent in the stone yard [*F*_(3,49)_ = 3.39, *p* = 0.025, see Figure 1]. Hens in pen two (*M* = 13.81, SEM = 3.81) spent longer in the stone yard than hens in pen three (*M* = 4.72, SEM = 1.74; *p* = 0.006) and pen four (*M* = 5.97, SEM = 1.44; *p* = 0.010), with a trend toward longer times than pen one (*M* = 8.81, SEM = 1.32; *p* = 0.088). There was a trend toward differing proportional times spent on the range between pens [*F*_(3,49)_ = 2.37, *p* = 0.082]. Time spent on the range was higher for hens from pen one (*M* = 9.53, SEM = 1.71) than pens two (*M* = 3.40, SEM = 2.22; *p* = 0.034) and three (*M* = 3.44, SEM = 2.12; *p* = 0.030). Hens from pen four spent an intermediate amount of time on the range (*M* = 5.80, SEM = 1.82), which did not differ from the other three pens. Accounting for pen as a random factor and attempts needed to induce TI as a fixed factor, the duration of TI was not associated with time in any of the four areas [barn: *F*_(1, 46.8)_ = 1.69, *p* = 0.200; wintergarden: *F*_(1, 46.3)_ = 0.58, *p* = 0.449; stone yard: *F*_(1, 43.1)_ = 2.94, *p* = 0.094; range: *F*_(1, 46.2)_ = 0.59, *p* = 0.448].

### Hippocampal Gene Expression

As expected, expression of LBR mRNA covaried with expression of PCNA [*F*_(1, 88.5)_ ≥ 264.7, *p* < 0.001] and DCX [*F*_(1, 75.8)_ ≥ 354.4, *p* < 0.001] over all models. Samples of the same HF subregion taken from the two hemispheres did not differ from each other in expression of either gene [PCNA: *F*_(1, 45.4)_ ≤ 0.296, *p* ≥ 0.589; DCX: *F*_(1, 47.5)_ ≤ 0.114, *p* ≥ 0.738]. Expression also did not differ between the rostral and caudal HF subregions for PCNA [*F*_(1, 62.0)_ ≤ 1.72, *p* ≥ 0.194] or DCX [*F*_(1, 51.3)_ ≤ 1.97, *p* ≥ 0.166] mRNA in any models.

### Ranging and Hippocampal Gene Expression

Proportional time spent in the barn did not correlate with PCNA mRNA expression across the whole HF [*F*_(1, 48.0)_ = 1.59, *p* = 0.214], though there was a trend toward an interaction with HF subregion [*F*_(1, 45.7)_ = 3.38, *p* = 0.073]. In the rostral HF, there was a trend toward a negative relationship between time in the barn and PCNA expression [*B* = −0.0030, SEM = 0.002, *F*_(1, 47.2)_ = 3.17, *p* = 0.082], with no relationship in the caudal HF [*B* = 0.0005, SEM = 0.001, *F*_(1, 40.2)_ = 0.33, *p* = 0.570]. PCNA expression was not associated with proportional time spent in the wintergarden [*F*_(1, 47.5)_ = 0.03, *p* = 0.866], and there was no interaction with subregion [*F*_(1, 46.1)_ = 0.72, *p* = 0.401].

Proportional time spent in the stone yard positively correlated with PCNA expression over the whole HF [*F*_(1, 46.6)_ = 6.54, *p* = 0.014, *B* = 0.0018, SEM = 0.003], and there was an interaction with subregion [*F*_(1, 44.7)_ = 4.57, *p* = 0.038]. While time in the stone yard positively correlated with PCNA mRNA in the rostral HF [*B* = 0.0146, SEM = 0.006, *F*_(1, 46.6)_ = 6.46, *p* = 0.014], there was no relationship in the caudal subregion [*B* = 0.0010, SEM = 0.033, *F*_(1, 34.4)_ = 0.12, *p* = 0.729].

**Figure 2:**
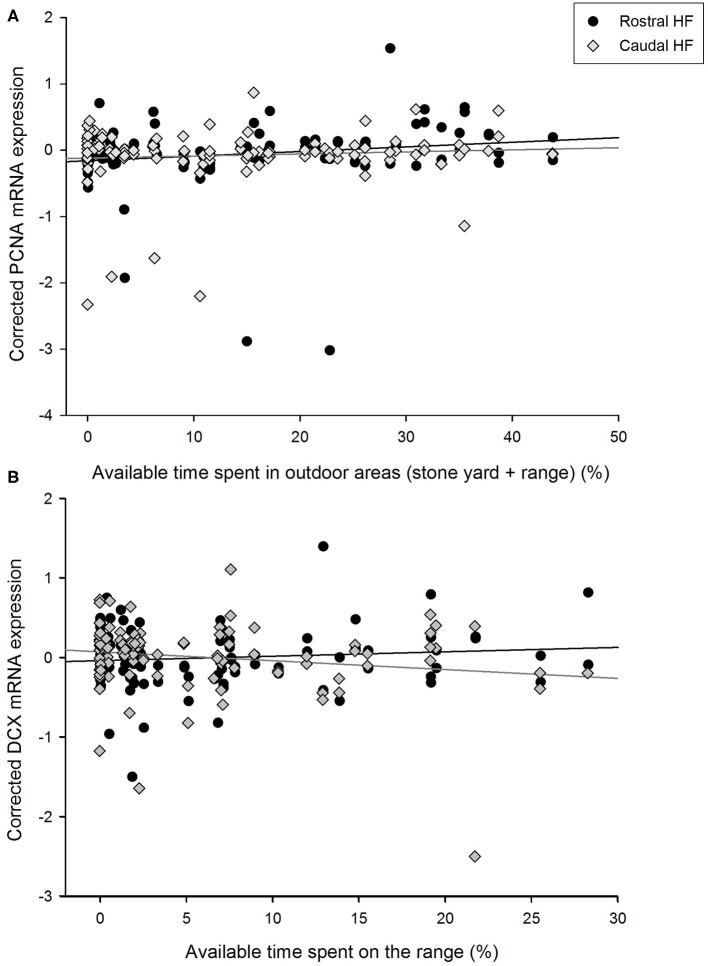
Relationships between the proportions of available time spent in outdoor areas by individual hens and corrected gene expression in the rostral and caudal subregions of the hippocampal formation. **(A)** PCNA expression in relation to the total percentage of time spent in outdoor areas (i.e., the stone yard + grassy range) by focal hens. **(B)** Doublecortin (DCX) expression in relation to the percentage of time that focal hens spent on the grassy range. Gene expression values are unstandardized residuals following correction for LBR expression, rostrocaudal subregion, sample, and pen, in linear mixed models.

The percentage of available time spent on the grassy range by hens did not correlate with their expression of PCNA mRNA in the HF as a whole [*F*_(1, 50.0)_ = 2.95, *p* = 0.092], but there was an interaction with rostrocaudal subregion [*F*_(1, 46.2)_ = 5.10, *p* = 0.029, Figure 2A]. Time on the range positively was positively associated with PCNA expression in the rostral HF [*B* = 0.0123, SEM = 0.006, *F*_(1, 45.6)_ = 4.10, *p* = 0.049] but not the caudal region [*B* = −0.0021, SEM = 0.003, *F*_(1, 41.8)_ = 0.46, *p* = 0.501]. As time in the stone yard and time on the grassy range correlated positively with each other and both related to PCNA expression, an association between their combined values (i.e., the total available time spent outdoors) and PCNA levels was also explored. Time outdoors was related to PCNA expression [*F*_(1, 50.6)_ = 6.75, *p* = 0.012, *B* = 0.0004, SEM = 0.002] and an interaction [*F*_(1, 44.7)_ = 6.64, *p* = 0.013] indicated that this association was again attributable to a positive relationship in the rostral HF [*F*_(1, 46.0)_ = 7.36, *p* = 0.009, *B* = 0.0092, SEM = 0.003], with no correlation in the caudal subregion [*F*_(1, 39.0)_ = 0.02, *p* = 0.895, *B* = −0.0002, SEM = 0.002].

Similarly, time spent on the range did not co-vary with hippocampal DCX expression over the whole HF [*F*_(1, 51.4)_ = 0.56, *p* = 0.456] but the difference in slopes between the rostral and caudal subregions was significant [*F*_(1, 54.8)_ = 4.72, *p* = 0.034, Figure 2B]. Time spent on the range was not associated with DCX expression in the rostral HF [*B* = 0.0062, SEM = 0.070, *F*_(1, 49.1)_ = 1.12, *p* = 0.296] but negatively correlated with DCX expression in the caudal HF [*B* = −0.0140, SEM = 0.007, *F*_(1, 53.2)_ = 4.09, *p* = 0.048]. DCX expression was not associated with time in any other area [barn: *F*_(1, 49.1)_ = 0.02, *p* = 0.896; wintergarden: *F*_(1, 49.2)_ = 0.11, *p* = 0.744; stone yard: *F*_(1, 50.3)_ = 1.25, *p* = 0.270], nor did these parameters interact with HF subregion [time in barn^*^subregion: *F*_(1, 51.0)_ = 0.17, *p* = 0.679; time in wintergarden^*^subregion: *F*_(1, 51.1)_ = 0.26, *p* = 0.613; time in stone yard^*^subregion: *F*_(1, 50.9)_ = 0.96, *p* = 0.332].

Lastly, the mean number of daily transitions between the four areas made by individual hens (a crude proxy for activity levels) did not correlate with expression of hippocampal PCNA [*F*_(1, 47.1)_ = 1.25, *p* = 0.261], though there was a trend toward an interaction with subregion [*F*_(1, 44.7)_ = 3.22, *p* = 0.080], with the slopes of the relationships tending in opposite directions [rostral: *B* = 0.0025, SEM = 0.002, *F*_(1, 45.7)_ = 1.64, *p* = 0.206; caudal: *B* = −0.0007, SEM = 0.001, *F*_(1, 41.0)_ = 0.60, *p* = 0.443]. DCX expression was not associated with number of transitions [*F*_(1, 49.0)_ = 0.01, *p* = 0.915; transitions^*^subregion *F*_(1, 51.0)_ = 0.03, *p* = 0.855].

### Tonic Immobility and Hippocampal Gene Expression

**Figure 3:**
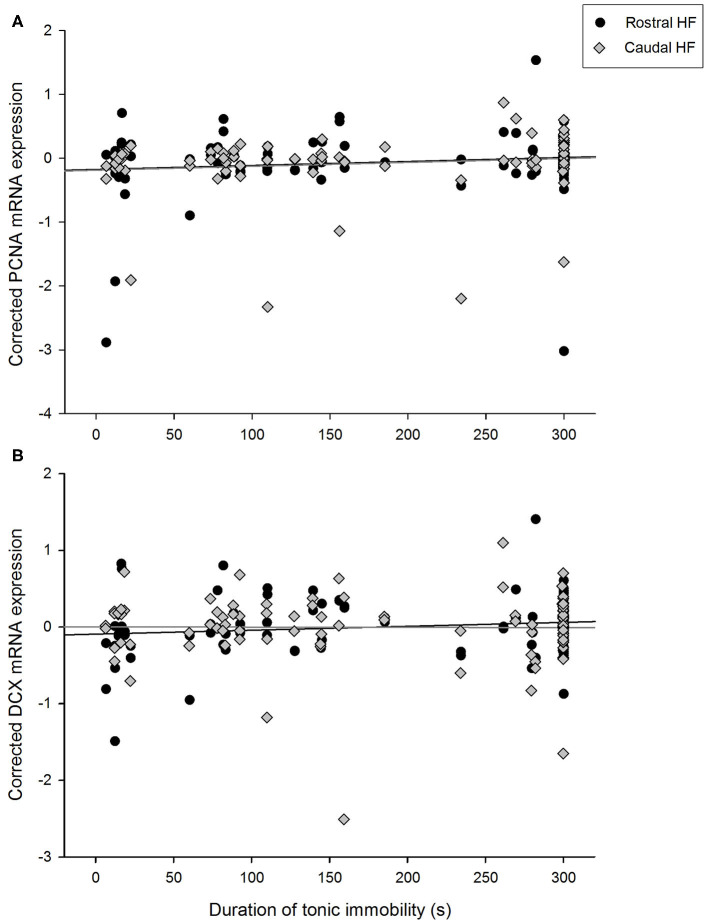
Relationship between durations of tonic immobility (seconds) for individual hens and residual expression of **(A)** PCNA and **(B)** doublecortin (DCX) in rostral and caudal subregions of the hippocampal formation, after correcting for LBR expression, rostrocaudal subregion, sample, and pen in linear mixed models.

Duration of TI positively correlated with expression of PCNA over the whole HF [*F*_(1, 45.0)_ = 5.60, *p* = 0.022] and did not interact with rostrocaudal subregion [*F*_(1, 40.9)_ = 0.43, *p* = 0.516, Figure 3A]. PCNA expression did not differ between hens requiring one, two or three attempts to induce TI [*F*_(2,39.8)_ = 1.46, *p* = 0.245], nor did number of attempts interact with subregion [*F*_(2,40.5)_ = 0.83, *p* = 0.443]. Conversely, hippocampal DCX mRNA expression was not associated with TI duration [*F*_(1, 47.4)_ = 0.68, *p* = 0.412] and there was no interaction with rostrocaudal subregion [*F*_(1, 49.2)_ = 0.96, *p* = 0.092, *p* = 0.333, Figure 3B]. DCX expression did not differ with attempts to induce TI [*F*_(2,46.6)_ = 0.97, *p* = 0.386], and there was no interaction with subregion [*F*_(2,46.6)_ = 1.47, *p* = 0.241].

To verify that proportions of time spent outdoors and durations of TI explained independent proportions of the variance in PCNA expression, they were included as covariates in the same model. TI duration continued to co-vary with PCNA expression throughout the HF [*F*_(1, 43.2)_ = 4.39, *p* = 0.042], and PCNA expression did not differ with the number of induction attempts [*F*_(2,39.5)_ = 1.34, *p* = 0.273]. Proportional time in outdoor areas co-varied with overall PCNA expression [*F*_(1, 47.0)_ = 5.72, *p* = 0.021], and the interaction between time outdoors and HF subregion remained significant [*F*_(1, 42.1)_ = 6.01, *p* = 0.018].

## Discussion

This study constituted an early exploration of the associations between individual differences and hippocampal plasticity in domestic chickens. Within the sampled flock of laying hens, individual differences in durations of TI were not correlated with time spent in any area of the housing system. Differences in TI have previously been reported between ranging sub-groups (69, 70), but another RFID-tracking study also failed to observe a relationship at the individual level (71). In the present sample, TI and use of the outdoor areas each explained separate portions of the total variance in expression of proliferative marker PCNA. The findings therefore support the existence of two independent relationships that link these behaviors to proliferation in the HF, each partially consistent with the hypothesized mechanisms.

Durations of TI positively correlated with expression of proliferative marker PCNA over the whole HF, suggesting that fearful, reactive hens have a higher level of hippocampal proliferation. This association is consistent with the predicted relationship between AHN and coping style. However, reactive hens would be expected to be less exploratory, but the associations between proliferation in the rostral HF subregion and proportional time spent in the furthest areas from the barn (the outdoor stone yard and range) were also positive. These subregional relationships are therefore more consistent with the predicted stimulatory effect of ranging experience on hippocampal cell proliferation. DCX expression, indicative of neuronal differentiation, displayed a generally different pattern from AHN cell proliferation: it negatively co-varied with proportion of time spent on the range, but only in the caudal HF. We will discuss each of the findings separately below.

### Hippocampal Gene Expression and Tonic Immobility (Coping Style)

Both higher basal levels of hippocampal proliferation and longer durations of TI are traits characteristic of individuals exhibiting a reactive (or passive) behavioral strategy/coping style (36, 37). Individual differences in proliferation, but not survival, have been positively related to the degree of freezing vs. locomotion displayed by rats in a novel environment (37). Supporting a causal contribution of new cells to reactivity, mice with experimentally-suppressed neurogenesis freeze less than wild-type mice when faced with a novel environment and stimulus during contextual fear conditioning (72). Reactive individuals also display enhanced behavioral flexibility (22), and hippocampal neurogenesis is necessary for flexible behavior during learning tasks (39, 40). It is theorized that adult-born neurons promote the erasure of previously learned associations, in order to minimize proactive interference and facilitate the acquisition of novel associations (73). Adult-born neurons have also been demonstrated to inhibit the activity of mature granule cells under conditions of novelty and anxiety (74, 75). As such, AHN may form part of the intrinsic mechanism which links individual differences in cognitive flexibility to those in behavioral responses to challenge. A higher level of proliferation may translate to a relatively higher number of surviving new neurons under certain conditions (76), but current research does not indicate how proliferating cells may exert functional effects prior to maturation and integration. A corresponding relationship between TI and expression of DCX would therefore be expected, and the absence of such a correlation may relate to the influence of environmental factors on neuronal differentiation, or to a methodological explanation, each discussed below. Furthermore, though reactive individuals are often less exploratory (16, 17), no relationship between freezing (TI) and ranging existed for the sampled flock. Previous studies in hens have explicitly linked behavioral flexibility, but not fearfulness, to ranging tendencies (26, 27). It may be that other dimensions of personality, such as sociability (77), are also influential determinants of ranging behavior, and obscure a simple relationship with reactivity.

### Ranging Experience and PCNA Expression

We also observed a significant positive relationship between ranging outside and PCNA expression, and this was specific to the rostral HF. Based on neuroanatomy, the dorsal rodent and rostral avian regions are hypothesized to be homologous, while the caudal avian HF is hypothesized to be homologous to the ventral rodent hippocampus (63). As TI durations were not associated with ranging in terms of the relative time spent in any area (internal or external), the relationship between ranging and PCNA expression is unlikely to relate to coping style. Instead, it may reflect the influence of ranging experience on hippocampal plasticity. In the rostral HF, time spent in both outdoor areas (the stone yard and grassy range) was positively associated with expression of *PCNA*. This relationship may be attributable to the stimulatory effect of factors including environmental complexity and exercise on hippocampal proliferation, as such experiences have been observed to preferentially modulate AHN in the dorsal rodent HF (48, 50, 78).

While the multi-tier barn interior comprises a complex, three-dimensional environment, all hens necessarily spent a substantial proportion of their time there: during the night and at other times that the additional areas were closed. Moreover, individual hens remained within the barn for a minimum of ~12% of the time that all areas were open, and it was the only location wherein certain key resources, including feed and nest boxes, were provided. Therefore, while the barn interior likely already comprised a cognitively challenging environment that could be considered enriched, this experience was shared by all birds. The wintergarden also provided resources in the form of drinkers and perches, and perhaps represented an extension of the barn in that it was used by all but one hen. In contrast, the lower proportion of hens that also regularly ventured farther afield, into the uncovered stone yard and range, effectively had a larger home range. This may entail maintenance of a larger mental map, while presenting greater navigational challenge to return to the resources provided inside. Size of the home range positively predicts hippocampal plasticity across species of rodent [reviewed in (79)], and the variety of territory coverage by individual mice roaming a complex home environment was strongly correlated with AHN (28). In birds, AHN rates are higher in migratory than non-migratory subspecies (of white crowned sparrow, *Zonotrichia leucophrys*) (80) and are stimulated by spatial-cognitive demand in experimental settings (57, 81), An increase in HF proliferation was observed following the storage and retrieval of food caches by Marsh tits (*Poecile palustris*) (81), making the spatial-cognitive challenge of ranging outside a likely contributor to the observed relationship with PCNA expression. While environmental enrichment has been found to upregulate numbers of proliferating cells in mice (78, 82–84), physical activity is perhaps a more robust driver of expansion of the precursor cell pool (42, 76). There was a trend for the number of transitions that hens made between areas to predict rostral PCNA expression, which may also point to a similar relationship between exercise and proliferation in chickens. However, as it was not possible to measure the individual distances traveled within each area, this measure provides only a crude proxy, and future experimental work will be needed to establish such an association.

### Ranging Experience and DCX Expression

Given that the stimulation of proliferation by a positive experience such as exercise leads to a subsequent increase in the number of surviving new-born neurons (76), the lack of corresponding positive relationships between time in the outdoor areas and rostral expression of *DCX* is also surprising. This finding further conflicts with the robust effect of enrichment on later stages of AHN (41, 42). Moreover, in the caudal HF, time spent on the grassy range alone correlated negatively with *DCX* expression. Downregulation of AHN consistently occurs following the experience of stress, and the ventral hippocampus in rodents (85, 86) and the caudal HF in laying hens (61) are known to be particularly sensitive. As certain forms of stress have a greater negative influence on the later survival of young neurons (87–89), it is possible that this factor is responsible for the decoupling of relationships with PCNA and DCX expression. The observed negative relationship between time on the range and caudal DCX expression implies that, while outdoor visits provide further environmental complexity (and possibly exercise), hens that spend more time on the range also experience more stress. This association is perhaps related to the consistent finding that many hens provided with outdoor access choose not to range (4–7). Indeed, the general assumption that the range represents an exclusively positive environment has not been demonstrated.

There is some evidence to suggest that enrichment may be stressful even within controlled laboratory settings. One study found that enriched housing including running wheels upregulated DCX immunoreactivity and mRNA expression in the dorsal mouse dentate gyrus, while suppressing levels in the ventral region (49). Housing domestic pigeons in an enriched environment has also been observed to increase the number of DCX-expressing neurons in the HF (rostral and caudal HF were not distinguished), while simultaneously increasing average durations of TI (56). Though a group effect was observed, the authors found no correlation between TI times and cell numbers on an individual level. Further investigation into whether some general aspects of enrichment, perhaps relating to the cognitive challenge, are intrinsically associated with stress may therefore be warranted.

Beyond laboratory enrichment, ranging outdoors may expose hens to unpredictable sources of stress, such as adverse weather conditions and sightings of predators. Individual range use was previously positively correlated with the CORT response to handling and flightiness to avoid a human (71), which may indicate greater anxiety. As time in the stone yard was not negatively associated with DCX expression, the characteristics which distinguish the range itself may be particularly stressful. Both areas were uncovered, potentially exposing hens to rain and sightings of aerial predators, but weather conditions such as wind may be more salient on the large, open range than in the smaller, fenced stone yard. Contact with soil is also associated with exposure to a greater burden of parasites (90), which may be a source of immune-stress. Perhaps due to extensive cover in the ancestral environment of red junglefowl, hens show a collective preference for shelter (91), whereas use of the open range entails being exposed. Over multiple commercial farms, the number of birds using an outside range correlates positively with the amount of tree cover provided (92), while addition of tree cover or shelters increases use of the range (93). The range in the present study was relatively barren and did not contain trees or other forms of shelter. Consistent with the sampled hens spending less time on the range than in other areas, nearest-neighbor-distance is generally observed to increase with increasing distance from the barn (2). This lack of proximity to conspecifics may be stressful, due to greater perceived predation risk or social isolation. On the other hand, as frequently ranging hens choose to be less close to their conspecifics (77), it is also possible that hens visit the range to escape social conflict with flock mates. In this case, the experience of stress would drive visits outside. However, we would therefore also expect to see an association with coping style, meaning this explanation is probably not consistent with the lack of correlation between ranging and TI.

In mice, neuronal survival to the point of maturation may be promoted specifically within the dorsal hippocampus by environmental enrichment (78, 84). Recent research in laying hens indicates that neuronal differentiation may be suppressed preferentially at the caudal pole (61) or over the whole HF (62) by different sources of chronic stress. If spending time outside is indeed a stressful experience for hens, then the stimulatory influence of cognitive stimulation may have counteracted the suppressive effect of stress in the rostral subregion, leaving an observable negative relationship only at the caudal pole. In mice, a combination of experimental stress and cognitive stimulation in the form of maze learning led to a preferential reduction in AHN in the ventral subregion (51). Such an interaction could explain the lack of a positive relationship between time spent outside and (rostral) DCX expression. In the case of proliferation, some studies have also found environmental enrichment to elevate levels specifically within the dorsal mouse hippocampus (84), while others have noted an increase in both subregions (78). Proliferating cell numbers may be reduced most severely in the ventral subregion by chronic stress (85), or be suppressed uniformly (84). It is therefore difficult to conclude whether an influence of stress on proliferation in the caudal HF may have contributed to the rostral-specific nature of the association between outdoor ranging and PCNA expression in the present study.

### Methodological Considerations

It is important to note that, while transcription of the DCX gene has been demonstrated to be a valid proxy for the effects of running on neuronal differentiation in mice (49), this association has yet to be verified in birds. Unlike in the mammalian brain, adult neurogenesis is not restricted to a single subdivision of the avian HF (equivalent to the dentate gyrus), meaning it is not possible to micro-dissect a particular substructure or to use a control gene specific to its cellular population (as with Prox1 for granule cells) for normalization. Previous research has noted background expression of DCX mRNA in non-neurogenic subdivisions of the mouse hippocampus, with levels unresponsive to running exercise (94). Though the majority of the avian telencephalon is neurogenic, low-level transcription of DCX in other types of HF cell, such as mature neurons undergoing dendrite-remodeling (95), might obscure correlations with expression of the marker by differentiating immature neurons. This issue of background expression relates specifically to the use of DCX as a marker, meaning our results for PCNA expression may be more reliable. Overall, while our findings suggest interesting relationships between behavior and AHN in domestic chickens, they would need to be validated using standard morphological techniques to quantify neurogenesis. The small effect sizes observed may reflect a complex interaction between the multiple internal and external factors which relate to AHN, but could also be linked to post-transcriptional processes which complicate the relationship between mRNA and protein levels (96). The specificity of such effects to the HF must also be confirmed by quantifying AHN in a control region of the telencephalon. Given that we are still working to establish the precise boundaries between the rostral/caudal subregions in our wider research, and this work therefore constitutes an early dataset that is building toward a better understanding of this hippocampal subdivision in birds.

## Conclusion

To conclude, individual differences in time spent on the free range and durations of TI in a commercial laying hen flock both positively correlated with cell proliferation in the HF (time on the range only in the rostral half), but were not related to each other. As found in other species, reactive hens had higher basal PCNA expression in the hippocampus, while exercise and enrichment through ranging were positively associated with PCNA expression in the rostral HF. Hippocampal proliferation thus most likely reflects both personality in terms of behavioral strategy/coping style and the influence of experience. On the other hand, expression of neuronal differentiation marker DCX in the caudal HF is negatively related to ranging experience. As the caudal HF may be preferentially sensitive to stress, it is thus possible that some aspects of ranging are both stimulating and stressful at the same time. However, this effect needs to be confirmed. Overall, individual differences in behavior are reflected in hippocampal plasticity, but probably for a number of different reasons.

## Data Availability Statement

The datasets generated for this study are available on request to the corresponding author.

## Ethics Statement

Experimental use of the animals was approved by the Bern Kantonal Authority (BE-46/16) and the Animal Welfare and Ethical Review Body at Newcastle University (Project ID #549).

## Author Contributions

EA collected tissue, conducted the molecular laboratory work, and drafted the manuscript. MT conceived of and designed the ranging study. BV, SV, and SG-H analyzed the ranging data. TS, TB, JG, and VS coordinated the AHN study. All authors gave final approval for publication.

## Conflict of Interest

The authors declare that the research was conducted in the absence of any commercial or financial relationships that could be construed as a potential conflict of interest. The reviewer PT declared a past co-authorship with the author SG-H to the handling editor.
